# Routine measurement of satisfaction with life and treatment aspects in mental health patients – the DIALOG scale in East London

**DOI:** 10.1186/s12913-020-05840-z

**Published:** 2020-11-09

**Authors:** Franziska Mosler, Stefan Priebe, Victoria Bird

**Affiliations:** 1 Unit for Social and Community Psychiatry, Institute of Population Health Sciences, Queen Mary University of London, London, UK; 2 East London NHS Foundation Trust, London, UK; 3 Present address: Unit for Social and Community Psychiatry, Newham Centre for Mental Health, London, E13 8SP UK

**Keywords:** DIALOG, Secondary mental healthcare, Routine outcome measurement, Quality of life, Treatment satisfaction

## Abstract

**Aims:**

The DIALOG scale has been implemented as a routine patient outcome and experience measure (PROM/PREM) in a mental health trust in East London since 2017. The resulting healthcare dataset was used to estimate satisfaction with life and treatment aspects over time and factors associated with it.

**Methods:**

Variables available from the Trust were DIALOG items, service level, clinical and basic demographic data. Data was extracted in February 2019. Data is described using a range of descriptive statistics and looking at the subgroups: treatment stage, diagnosis, service type. Predictors for average DIALOG scores across patients was explored with clustered linear regression models. A fixed effect model was chosen to estimate the impact of clinical and service related variables on patient’s average DIALOG scores over time. Sensitivity analyses with the whole data set and complete cases were carried out.

**Results:**

Of the original 18,481 DIALOG records 12, 592 were kept after data cleaning (5646 patients). The average DIALOG score was 4.8 (SD 1.0) on the 7-point scale. Average satisfaction with life aspects (PROM) was 4.65 (*SD* 1.1) and with treatment aspects (PREM) was 5.25 (*SD* 1.17). Across all 11 items, “job situation” scored lowest (mean 4.05) and “meetings with professionals” highest (mean 5.5). Satisfaction for all items increased over time (average increase 0.47). The largest increase was in “mental health” (0.94) and the smallest in “family relationships” (0.34).

**Conclusions:**

Patients in mental healthcare services were “fairly satisfied” in both life and treatment aspects with improvements seen over time. These results will act as a benchmark for clinical services currently implementing DIALOG across the UK and inform local service developments.

## Background

Patient-reported outcome and experience measures (PROM/PREM) have been developed to include the patient perspective in healthcare delivery and quality improvement [1]. In mental healthcare subjective quality of life (SQOL) is a useful PROM as improving quality of life has been stated as its specific aim [2–4]. Treatment satisfaction on the other hand is a PREM that gives insight into process and quality of mental healthcare [5].

People with diagnoses of mental health disorders are reported to have lower quality of life than the general population [6]. Cross-sectional studies have struggled to identify consistent associations between subjective quality of life and social and clinical variables. In studies to date, symptoms of anxiety and depression as well as “number of unmet needs” are reported most consistently to have large negative impact on SQOL [3, 7]. Other findings have been that patients in the community typically have better SQOL that than those staying in institutions [3, 8]. Studies tracking SQOL over time have been inconsistent on whether improvements occur [9].

Treatment satisfaction is generally rated high by those using mental healthcare services but varies depending on specific treatment aspects covered [10–12]. Socio-demographic characteristics such as age or gender have shown weak or inconsistent associations with treatment satisfaction; whereas clinical and care aspects such as unmet needs or adequate care environments are more informative [5].

DIALOG is a validated patient reported outcome and experience measure (PROM/PREM). The scale complies with the requirements for routine outcome assessment in mental health services as suggested by Slade [7]. Patients are asked to rate their satisfaction with each of eight life domains (mental health, physical health, job situation, accommodation, leisure, partner/family, friendship, personal safety) and three treatment aspects (medication, practical help, meetings with healthcare professionals). The 7-point scale ranges from “totally dissatisfied” to “totally satisfied” with the value 4 representing a neutral “in the middle”. DIALOG combines outcome measurement with treatment planning and discussion that is immediately relevant to patients, avoiding additional burden for patient and services that normally hamper routine implementation of such measures [13, 14].

After conducting the initial trial for effectiveness of DIALOG as a therapeutic intervention [15], assessment of the psychometric qualities of the DIALOG scale confirmed its usability as outcome measure in routine community mental health care [13]. Following that, the intervention was further refined into DIALOG+ and shown clinically effective in two randomised controlled trials in community mental health teams in East London [16] and medium-secure services in South England [17], respectively, as well as a small pilot study in Austria [18]. Since 2017, DIALOG+ has been implemented within East London NHS Foundation Trust (ELFT) as part of a new care plan approach and to collect patient outcome and treatment experience data; the latter will be the focus of the paper. Founded in 2000 in East London, the Trust now provides mental health care to a population of 1.3 million people across nine boroughs in and around London.

The resultant dataset presents a unique opportunity to explore subjective quality of life and treatment satisfaction in a local population attending adult mental health services. Furthermore, it provides reference scores for routine evaluation of other Trusts implementing the scale across the UK.

The aim of this study was to explore DIALOG scores in mental health patients in East London. Specific objectives were to: 1) estimate satisfaction across life (PROM) and treatment aspects (PREM) 2) explore demographic, service, and clinical factors associated with satisfaction 3) explore change in satisfaction over time, and 4) explore demographic, service, and clinical factors linked to change over time.

## Methods

### Design & aim

This service evaluation sought to establish the standards, i.e. average scores, that can be expected when using DIALOG as a routine outcome measure trust-wide.

### Setting & participants

Since implementation in 2017 every patient in ELFT should be completing the DIALOG scale when entering or leaving services, as well as during regular intervals as part of the care planning meetings with clinicians. Staff members receive mandatory training in completing the DIALOG+ intervention and can either enter scale responses directly onto the electronic patient record RiO or collect them on printouts initially. The study population should be a representative and near complete sample of adult patients seen by the Trust from January 2017 to February 2019. Additional data from 2016 was also available from a number of pilot services within the Trust, and was therefore included.

### Ethics

As per Health Research Authority guidance consent was not required and permission to access and use anonymous data as part of a service evaluation was gained from ELFT’s Governance and Ethics Committee for Studies and Evaluations.

### Procedure

A request was submitted to the data warehouse to obtain routinely collected, anonymised data on the following variables:

1. DIALOG scale (11 items, 7-point Likert scale), “additional help needed” (yes/no)
2. Service level data: team, directorate, stage of treatment, care programme approach (CPA) status, duration with the Trust
3. Clinical data: Health of the Nation Outcome Scales (HoNOS), ICD-10 code (primary diagnosis only), cluster
4. Demographics: gender, age (18-65 years), ethnicity

### Analysis

The dataset was managed and analysed using STATA 15 (StataCorp, 2017). Data is described using a range of descriptive statistics and looking at the following subgroups: treatment stage, diagnosis, service type.

Predictors for average DIALOG scores across patients were explored using clustered linear regression models. Two separate models were built for the PROM component of the measure that consists of eight life aspects and the PREM component that includes the three treatment aspects. For the cohort of patients that have more than one DIALOG entry on the electronic patient record, time series analysis was conducted to explore trends. A fixed effect model was chosen to estimate the impact of clinical and service related variables on patients’ average DIALOG scores over time. The dataset was treated as an unbalanced panel with a relative large number of observations and short time dimension. All available demographic, clinical, and service variables were assessed in multivariate models, selecting covariates based on *p* values < 0.01.

#### Data cleaning

The values for individual clinical teams, ICD diagnoses, and directorates were re-grouped into broader categories:

The 208 clinical teams within ELFT were grouped into two service types to allow for comparison: “community services” included community mental health teams (CMHTs), early intervention, psychology, OT, art therapies, enhanced primary care, learning disability, and older adults (139 teams); and “acute services” included inpatient, home treatment, and perinatal teams (69 teams).

Based on recorded ICD-10 F codes, four categories, “F2”, “F3”, and “Other” were created. The “Other” category encompassed mainly patients with F1, 4, and 6 diagnoses, but all the other nine codes were represented.

Trust directorates were condensed by combining the three values “Bedfordshire”, “Luton”, “Luton & Bedfordshire” into one overarching category.

#### Time points

In order to create meaningful time intervals while retaining maximum data, only the first DIALOG entry was kept when they were:

- on the same day,
- less than7 days apart in inpatient services
- less than 30 days apart in community teams

However, if patients moved between two services or clinicians entered the session as a different treatment stage, the interval could be closer than 7/30 days.

#### Missing data

As part of the data cleaning only observations with less than 20% of the 11 DIALOG items missing were included to calculate the overall mean for the DIALOG scale – effectively using mean imputation to address missing data in this variable.

#### Sensitivity analysis

In order to address any potential bias introduced through the data cleaning process, sensitivity analyses including the “whole data set” and “complete cases only” (i.e. all 11 items completed) were carried out.

## Results

### Demographic, clinical, and service -level characteristics

There were a total of 18,481 DIALOG records from 7763 patients recorded within the time span of 3 years. Patient and service characteristics are summarised in Table 1. Patients were predominantly male (52%), white (47%), and on average 38 years old. Patients were most often given a diagnosis within the ICD-10 F2 category (33%) and more than a third of patients did not have any recorded diagnosis. The average HoNOS score was 14.1 and 18% of records came from patients with a legal status, i.e. those in services under the Mental Health Act. Two thirds of patients were on the CPA and in half the cases clinicians had indicated that DIALOG scale was completed as part of the CPA meeting.

**Table 1:** Demographic, clinical, and service level characteristics of patients

	n	Mean *(SD; Min -Max)*
**Gender** *(male)*	7763	52.2%
**Age**	7763	38.3 (12.4; 18–65)
**Ethnicity**	7763	
Asian	1651 (21.3%)	
Black	1599 (20.6%)	
White	3673 (47.3%)	
Other Ethnic Group	460 (5.9%)	
Not Known	380 (4.9%)	
**HoNOS**	6806	14.1 *(7.5; 0–45)*
**ICD diagnosis**		
Organic disorders -F0	7 (0.1%)	
Disorders due to psychoactive substances -F1	215 (2.8%)	
Schizophrenia & related disorders -F2	2543 (32.8%)	
Mood disorders -F3	1118 (14.4%)	
Neurotic, stress-related & somatoform disorders -F4	392 (5.1%)	
Behavioural syndromes assoc. with physiological disturbances and physical factors -F5	46 (0.6%)	
Disorders of adult personality and behaviour -F6	431 (5.6%)	
Mental retardation -F7	23 (0.3%)	
Disorders of psychological development -F8	38 (0.5%)	
Behavioural and emotional disorders with onset usually occurring in childhood and adolescence -F9	14 (0.2%)	
Unspecified mental disorder -F99	2 (0.03%)	
Missing	2934 (37.8%)	
**Super Cluster**		
Psychotic	2150 (27.7%)	
Non-Psychotic	1193 (15.4%)	
Organic	12 (0.1%)	
Missing	4408 (56.8%)	
**Client on Care Planning Approach (CPA)** *(Yes)*	12,668 (68.6%)	
**Duration with Trust** *(Days)*	18,478 *(3 missing)*	2332.2 *(2112.7; 0–36,574.0)*
**Directorate**		
Bedfordshire	2217 (12.0%)	
City &Hackney	3511 (19.0%)	
Forensic	1712 (9.3%)	
Luton	2128 (11.5%)	
Newham	3585 (19.4%)	
Tower Hamlets	4332 (23.4%)	
Bedfordshire &Luton	996 (5.4%)	
**Inpatient**		
Inpatient	2934 (15.9%)	
Home Treatment Team (HTT)	207 (1.1%)	
Other	15,340 (83.0%)	
**Service Classification**		
Community Mental Health Team (CMHT)	9149 (49.5%)	
Early Intervention	3083 (16.7%)	
Psychology, Arts, Occupational Therapy	1548 (8.4%)	
Inpatient, Liaison	1814 (9.8%)	
HTT, Crisis	437(2.4%)	
Primary Care	158 (0.9%)	
Older Adults	39 (0.2%)	
Learning Disabilities	420 (2.3%)	
Perinatal	109 (0.6%)	
Forensic Inpatient	1036 (5.6%)	
Forensic Community	688 (3.7%)	
**Legal Status** *(Yes)*	3303 (17.9%)	
**Treatment Stage**		
Assessment	5839 (31.7%)	
Review	12,208 (66.3%)	
Discharge (CPA & Trust)	378 (2.1%)	
Missing	56 (0.3%)	
**Dialog completed as part of CPA**		
Yes	10,283 (55.6%)	
Assessment & Plan (A&P)	10 (0.05%)	
Ongoing A&P	29 (0.2%)	
Other Review &Plan	7 (0.04%)	
Discharge & Review	2 (0.01%)	
Missing	8150 (44.1%)	

The vast majority of patients were seen in community teams (83%) with an even spread across the four main borough directorates. 208 teams recorded DIALOG responses; half of those were done by CMHTs and another fifth by Early Intervention services.

In terms of treatment stages, two thirds of records were reviews, another 32% initial assessments and only 2% at discharge. At the time of completing the DIALOG scale, patients had been with the Trust for average of 6.3 years.

### DIALOG scores

Across the whole data set, 2.6 items out of eleven were missing on average within DIALOG records. The amount of missing data for individual items ranged from 17% for “mental health” to 31% for “practical help”.

The cleaned data set contained 5646 individual patients with 12,592 unique observations overall. The average DIALOG score was 4.8 (*SD* 1.0) which equates to “fairly satisfied” anchor on the scale. Separated out by PROM and PREM, across the whole data set satisfaction with life aspects was 4.65 (*SD* 1.1) and satisfaction with treatment aspects was 5.25 (*SD* 1.17).

#### DIALOG scores by item

**Fig. 1:**
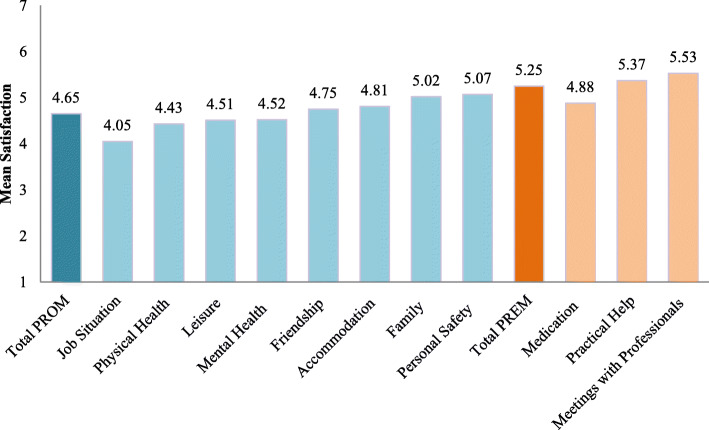
Average satisfaction by DIALOG item

The average satisfaction scores of individual PROM and PREM items are shown in Fig. 1 (Table 2). Across life aspects patients were the least satisfied with their “job situation” (4.05 = “in the middle”) and the most with “personal safety” (5.07 =“ fairly satisfied”). Of the three treatment items “medication” was rated lowest (4.88 = “fairly satisfied”) and “meetings with professionals” highest (5.5 =“ fairly” to “very satisfied”).

**Fig. 2:**
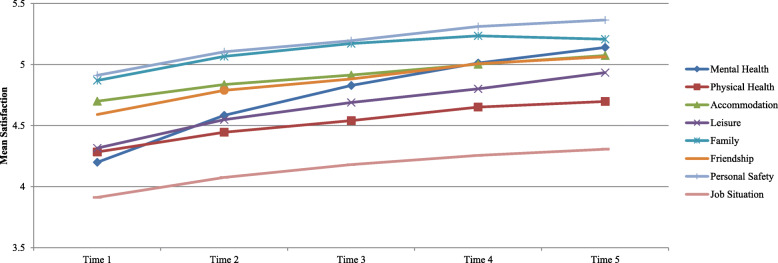
Change in average satisfaction with life aspects over time

**Table 2:** Satisfaction across life and treatment aspects (*n* = 12,592)

	Mean	SD	Missing
*Mean by observation*	4.81	1.01	–
*Total PROM*	*4.65*	*1.1*	
**Job Situation**	4.05	1.73	1263 (10%)
**Physical Health**	4.43	1.64	135 (1%)
**Leisure**	4.51	1.57	251 (2%)
**Mental Health**	4.52	1.71	91 (1%)
**Friendship**	4.75	1.57	434 (3%)
**Accommodation**	4.81	1.84	136 (1%)
**Family**	5.02	1.64	446 (4%)
**Personal Safety**	5.07	1.56	287 (2%)
*Total PREM*	*5.25*	*1.17*	
**Medication**	4.88	1.56	504 (4%)
**Practical Help**	5.37	1.4	1018 (8%)
**Meetings with Professionals**	5.53	1.31	442 (4%)

**Fig. 3:**
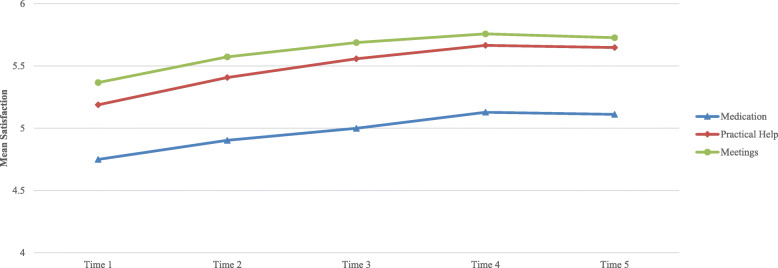
Change in average satisfaction with treatment aspects over time

Subgroup analysis based on treatment stage found that average satisfaction increases for all items between the sequential stages. Sensitivity analysis demonstrated no significant differences in average items scores across the three datasets.

#### Predicting DIALOG scores (clustered)

All available demographic, clinical, and service characteristics were included in the models. Four of the predictor variables had missing data: “Duration with the Trust” (0.15%), HoNOS (4.53%), treatment stage (0.28%), and ICD-10 F code (29.3%). The “SuperCluster” variable was excluded from the models due to high percentage of missing data (56.3%) and strong association with the F code variable.

##### PROM – subjective quality of life

The final model shown in Table 3 explained 15% of the variance of average quality of life. Factors associated with significantly higher satisfaction were being male, being treated under the Mental Health Act, being treated on a CPA, completing the DIALOG scale as part of a CPA meeting, attending services within the forensic directorate, and completing the scale during a review or discharge meeting rather than at assessment. Lower satisfaction was predicted by lower clinician-rated health and social functioning (HoNOS), as well as having a recorded diagnosis other than the schizophrenia spectrum.

**Table 3:** Predictors of average satisfaction with life aspects

Variables	β Coef	Std. Err.
**HoNOS**	−0.029*	(0.002)
**Legal Status** *(No* vs.*)*		
*Yes*	0.207*	(0.041)
**Gender** *(Female* vs.*)*		
*Male*	0.115*	(0.037)
**Is the patient on the CPA?**		
*Yes*	0.226*	(0.039)
**Completed as part of CPA?**		
*Yes*	0.211*	(0.031)
**ICD-10 Code** *(Schizophrenia & related disorders -F2* vs.*)*		
*Mood disorders -F3*	−0.260*	(0.044)
*Other*	−0.364*	(0.050)
**Directorate** *(Newham* vs.*)*		
*Forensic*	0.270*	(0.078)
**Treatment Stage** *(Assessment* vs.*)*		
*Review*	0.168*	(0.038)
*Discharge*	0.345*	(0.095)
**Constant**	4.659	(0.060)
***Observations***	8743	
***Number of Clusters***	3609	
***R-squared***	0.154	

* *p* < 0.01

##### PREM –treatment satisfaction

The model shown in Table 4 included eight variables that explained 8% of average treatment satisfaction in this population. The positive predictors were being male, on a CPA, and completing the DIALOG form as part of a CPA meeting, review or discharge; with discharge having the largest positive impact. Treatment satisfaction was negatively impacted by lower clinician rated functioning (HoNOS), being treated under the Mental Health Act, having a diagnosis other than schizophrenia and mood disorders, and being treated by services in Tower Hamlets or Luton& Bedfordshire compared to Newham.

**Table 4:** Predictors of average satisfaction with treatment aspects

Variables	β Coef	Std. Err.
**HoNOS**	−0.021*	(0.002)
**Legal Status** *(No* vs.*)*		
*Yes*	−0.267*	(0.059)
**Gender** *(Female* vs.*)*		
*Male*	0.140*	(0.039)
**Is the patient on the CPA?**		
*Yes*	0.236*	(0.043)
**Completed as part of CPA?**		
*Yes*	0.181*	(0.037)
**ICD-10 Code** *(Schizophrenia & related disorders -F2* vs.*)*		
*Other*	−0.150*	(0.051)
**Directorate** *(Newham* vs.*)*		
*Tower Hamlets*	−0.171*	(0.046)
*Bedford &Luton*	−0.251*	(0.049)
**Treatment Stage** *(Assessment* vs.*)*		
*Review*	0.225*	(0.043)
*Discharge*	0.401*	(0.107)
**Constant**	5.203	(0.064)
***Observations***	8743	
***Number of Clusters***	3609	
***R-squared***	0.082	

* *p* < 0.01

#### DIALOG scores over time

An average of two time points (SD 1.33; min-max: 1–13) were available per patient after the dataset was cleaned to retain only meaningful time intervals. However, the number of patients included in each additional time point decreased significantly, such that only 7% of patients (*n* = 394) had five records, therefore this was used as the cut off for change over time comparisons.

Average satisfaction between time point one to five improved by 0.47 (from 4.64 to 5.11), with PROMs improving marginally more than PREMs (0.50 vs 0.40). Satisfaction for all individual DIALOG items increased over time (see Table 5) and was robust to sensitivity analysis.

**Table 5:** Change over time in average satisfaction by DIALOG item

	Time 1	Time 2	Time 3	Time 4	Time 5	Change T1 to T5
*Total PROM*	*4.48*	*4.68*	*4.80*	*4.91*	*4.98*	*0.50*
**Mental Health**	4.20	4.58	4.83	5.01	5.14	0.94
*n*	*5595*	*3263*	*1959*	*1016*	*394*	
**Physical Health**	4.28	4.44	4.54	4.65	4.70	0.42
*n*	*5572*	*3250*	*1954*	*1015*	*393*	
**Accommodation**	4.70	4.84	4.91	5.0	5.07	0.37
*n*	*5576*	*3247*	*1956*	*1009*	*394*	
**Leisure**	4.32	4.55	4.69	4.80	4.93	0.62
*n*	*5505*	*3231*	*1940*	*1005*	*392*	
**Family**	4.87	5.07	5.17	5.24	5.21	0.34
*n*	*5431*	*3170*	*1904*	*988*	*386*	
**Friendship**	4.59	4.79	4.88	5.01	5.06	0.47
*n*	*5414*	*3168*	*1912*	*998*	*393*	
**Personal Safety**	4.91	5.10	5.20	5.31	5.37	0.45
*n*	*5477*	*3218*	*1939*	*1007*	*392*	
**Job Situation**	3.91	4.08	4.18	4.26	4.31	0.40
*n*	*5026*	*2954*	*1791*	*931*	*369*	
*Total PREM*	*5.09*	*5.28*	*5.41*	*5.50*	*5.48*	*0.40*
**Medication**	4.75	4.90	5.0	5.13	5.11	0.36
*n*	*5318*	*3175*	*1939*	*1003*	*390*	
**Practical Help**	5.19	5.41	5.56	5.67	5.65	0.46
*n*	*5123*	*3033*	*1839*	*949*	*372*	
**Meetings**	5.37	5.57	5.69	5.76	5.73	0.36
*n*	*5395*	*3188*	*1932*	*993*	*381*	

Figures 2 and 3 show how the amount of change over time differed across individual PROM &PREM items. The largest increases were seen in the “mental health” and “leisure activities” domains (0.94 and 0.62) moving them both to the “fairly satisfied” scale point. “Family relationships” improved the least with a 0.34 increase. “Job situation” was consistently scored as the lowest DIALOG item and “meetings with health professionals” as the highest across all time points

##### Subgroup analysis

There are differences in average life and treatment satisfaction across the three diagnostic subgroups with patients with a F2 code reporting the highest scores on both measures (4.9 and 5.4). Over time scores improved in all groups with patients in the “other” category making the most gains (Table 6).

**Table 6:** Change over time in average satisfaction scores by diagnostic group

	*n*	Time 1	Time 2	Time 3	Time 4	Time 5	Change T1 to T5
*Total Patients*		*3722*	*2404*	*1510*	*782*	*290*	
**Schizophrenia & related disorders -F2** *(n)*	*5348*	*2004 (35.5%)*	*1476 (44.9%)*	*992 (50.3%)*	*530 (52.0%)*	*196 (49.8%)*	
PROM		4.78	4.87	4.92	4.98	5.06	0.28
PREM		5.31	5.40	5.47	5.56	5.58	0.28
**Mood disorders -F3** *(n)*	*1750*	*860 (15.2%)*	*454 (13.8%)*	*248 (12.6%)*	*121 (11.9%)*	*46 (11.7%)*	
PROM		4.30	4.45	4.53	4.62	4.65	0.35
PREM		5.03	5.16	5.32	5.36	5.24	0.21
**Other** *(n)*	*1804*	*858 (15.2%)*	*474 (14.4%)*	*270 (13.7%)*	*131 (12.8%)*	*48 (12.2%)*	
PROM		4.09	4.33	4.52	4.75	4.75	0.65
PREM		4.82	5.07	5.30	5.49	5.22	0.40

In terms of service type, “acute services” had better average life satisfaction (4.8 vs. 4.6) but “community services” had higher treatment satisfaction (5.3 vs 5.1). Both service types improved over time on both measures (see Table 7). However, as above, the number of records reduced substantially, with time point five representing 3% of total records.

**Table 7:** Change over time in average satisfaction scores by service type

	Total	Time 1	Time 2	Time 3	Time 4	Time 5	Change T1 to T5
*Total Patients*		*5646*	*3286*	*1972*	*1020*	*394*	
**Community** *(n)*	*10,255*	*4615*	*2747*	*1623*	*803*	*280*	
*PROM*		4.45	4.66	4.78	4.84	4.89	0.44
*PREM*		5.12	5.31	5.46	5.53	5.50	0.39
**Acute** *(n)*	*2337*	*1031*	*539*	*349*	*217*	*114*	
*PROM*		4.59	4.79	4.90	5.17	5.20	0.61
*PREM*		4.97	5.14	5.16	5.38	5.44	0.47

#### Predicting DIALOG scores over time

From the available variables “duration with the Trust”, “treatment stages”, and “acute services” predicted the average DIALOG score for individual patients’ over time. As shown in Table 8, the overall explained variance as well as individual coefficients were small, in particular “duration” had no practically relevant impact on the scale. Progressive treatment stages increased satisfaction, whereas being in acute services reduced it slightly.

**Table 8:** Predictors of average DIALOG scores across individual patients

Variables	β Coef	Std. Err.
**Duration with the Trust**	0.000*	(2.49e-05)
**Treatment Stage** *(Assessment* vs.*)*		
*Review*	0.150*	(0.0193)
*Discharge*	0.226*	(0.0424)
**Service Type** *(Community* vs.*)*		
*Acute Service*	−0.087*	(0.0243)
**Constant**	4.129*	(0.0576)
***Observations***	*12,538*	
***Number of Patients***	*5635*	
***R-squared***	*0.033*	

*p < 0.01

## Discussion

### Summary of results

This analysis of routine healthcare data found that on average, patients in East London NHS Foundation Trust were “fairly satisfied” with treatment aspects (PREM) receiving higher scores compared to life aspects (PROM) throughout all time points. Both PROM and PREM scores increased over time. “Mental health” satisfaction scores increased most rapidly whereas “job situation” remained the lowest scoring item.

A number of patient, clinical, and service variables were identified as predictive of average PROM and PREM scores and overall changes in satisfaction over time. However, models remained poorly specified, indicating that important predictors were missing from the available dataset.

Subgroup analyses showed small differences in satisfaction between diagnostic groups & service types. Patients with an F2 diagnosis reported higher life and treatment satisfaction compared to other diagnostic groups. Patients seen by “acute services” had higher life satisfaction but lower treatment satisfaction compared to those seen in “community services”. Rate of improvement was largest for patients with “other” diagnoses (i.e. not F2 or F3) and those seen in “acute services”. Overall DIALOG scores also improved between the progressive treatment stages of initial assessment, review, and discharge.

### Strengths & Limitations

This exploration of routinely collected DIALOG data contributes to the growing evidence-base for the inclusion of subjective quality of life as a routine outcome measure [16–20]. The implementation of DIALOG as an outcome and experience measure allowed for insights across a near-complete local population of secondary care mental health patients beyond traditional research designs. Where previous studies focused on patients with psychosis seen in CMHTs, this is the first time DIALOG scores have been analysed across mental health conditions, healthcare settings, and over time.

Routine healthcare datasets come with limitations regarding what variables are available to answer research questions; for example, estimating the impact of physical comorbidities on DIALOG scores would be important from a clinical and service development perspective but that information was not available in the current dataset. Further, the value of healthcare datasets heavily depends on effective routine processes that collect valid and complete data. As these datasets are not created for the purpose of research, missing data can frequently outweigh observed values [21]. Therefore, it is a strength of this study that 70% of the whole dataset has been included in the final analysis and data loss was only partially due to missing values as we put additional restrictions on time intervals for records e.g. removing multiple entries that did not occur within the pre-specified time point.

As discussed in previous publications, validity of records is threatened by social desirability bias that might manifest when patients rate their satisfaction in front of their clinician [13], e.g. it is possible that the consistently high treatment satisfaction scores in this dataset are due to this bias. Experimental research into the impact of the patient-clinician relationship on life satisfaction ratings showed it to be significant but not consistent, unidirectional, or uniform across life domains [22]. Thus, even though this effect has to be considered when interpreting the data, it is unknown to what extent item ratings would be different.

### Comparison with literature

#### PROM – subjective quality of life

There is no previous research reporting on subjective quality of life in cross-sectional or longitudinal designs across the heterogeneous population attending secondary mental health services. However, there has been a smaller study of patients attending community mental health appointments that used a precursor of the DIALOG scale, which found similar average satisfaction ratings (between “mixed” and “mostly satisfied”) with small improvements over two follow up periods [23]. Focusing just on schizophrenia, a pooled analysis of 886 patients reported a mean improvement of DIALOG-related life satisfaction measures of 0.20 over periods ranging from 6 to 36 months [24]. This is similar to the average change we found in patients with a diagnosis categorised under ICD-10 F2.

Extensive research exists on factors predicting subjective quality of life, for example self-esteem, satisfaction with services [23], unmet needs [2], and symptom levels [25]. This study adds to this known literature by exploring more clinical and service characteristics as opposed to individual factors. However, from the available clinical and demographic variables findings were in line with previous research, for example, we found higher satisfaction with life aspects in patients with psychotic disorders compared to mood or neurotic diagnoses [25–28]. Results from experimental studies have suggested that this is because affective states inform and direct judgements on satisfaction [29].

Varied gender differences in SQOL have been found in a number of different mental health populations which is why it’s unusual that our analysis pointed to lower satisfaction in women across all SQOL items. Previous research on a large sample of patients with diagnosis of schizophrenia reported out of ten life aspects women were only less satisfied with their personal safety compared to men [30]. Gamma & Angst [31] included a large heterogeneous group of mental health patients (without psychosis) and found women to be less satisfied with their physical and mental health but not work, finances, or relationships in general.

#### PREM - treatment satisfaction

At this time there is no comparative data available from other studies regarding treatment satisfaction as measured by the DIALOG scale. In general, Hansson and colleagues [32] have argued absolute treatment satisfaction scores are more informative than any changes over time. Not only is treatment satisfaction just generally reported to be high within the literature, ceiling effects are also common suggesting continuous improvements are unlikely [33]. This is partially reflected in this dataset; the three treatment aspects improve from time points one to four but then plateau at time point five. Even though the reported regression model explained little of the variance, our predictors associated with lower treatment satisfaction such as being female and under compulsory treatment are supported by other studies [10, 11]. On the other hand, this dataset showed higher satisfaction scores for patients in the psychotic cluster but Blenkiron and Hammill [34] reported no differences between diagnostic groups, albeit using a different measure.

### Implications for research and practice

The main purpose of analysing DIALOG scores based on routine data is to set a point of comparison for future benchmarking. This will allow services to use their own DIALOG scores to highlight any individual areas to focus on. For example, any extreme deviation from these averages could be raised with clinical management teams for review of their treatment provision or referral criteria, ideally leading to more appropriate interventions for those people who require them.

Therefore, processes need to be set up on a local level which enable the organisation to use results of analysed data and develop relevant questions and variables for the routine dataset further whilst engaging in a feedback loop on data quality and validity. Further, strategies need to be developed to ensure the collection and analysis of routine data results in translation of knowledge into clinical practice [7, 35].

The limited specificity of routine care data can lead to misleading conclusions making more in depth research necessary in some areas [36]. In this case, based on the satisfaction ratings “mental health” seems to be a major concern when patients enter services but as this area improves in the longer term, “job situation” seems to remain problematic. These low satisfaction ratings for could be further investigated, for example whether a change in service provision could address this.

In the near future, more accurate data will become available as DIALOG will continued to be used over significant time spans for a larger cohort of patients. Additionally, more extensive data is currently available from electronic patient records and could be explored for trends and comparisons between subgroups relevant to local needs. In the more distant future, discussions around public access to outcomes from routine mental healthcare, as the IAPT programme has created, should be considered to improve transparency and develop effective healthcare [37].

## Conclusion

This analysis presented life and treatment satisfaction of patients in mental healthcare services as measured by the DIALOG scale, available for the first time from routine care data. The data suggested that on average patients were “fairly satisfied” in both aspects and that satisfaction improved over time. These results can contextualise research trial evidence and benchmark data from clinical services implementing DIALOG as an outcome measure or intervention. Additionally, tracking individual items over time, e.g. those consistently rated lower than average; can inform future service developments.

## Data Availability

The data that support the findings of this study are available on request from the corresponding author FM. The data are not. publicly available due to them containing information that could compromise patients’ privacy.

## Abbreviations

CMHT: Community Mental Health Team
CPA: Care Programme Approach
ELFT: East London NHS Foundation Trust
HoNOS: Health of the Nation Outcome
ICD-10: International Classification of Diseases 10th Revision
PREM: Patient Experience Measure
PROM: Patient Outcome Measure
SQOL: Subjective Quality of Life
